# Genome sequences of 11 *Alkalihalobacillus clausii, Bacillus safensis,* and *Escherichia coli* bacteriophages isolated from bovine rumen and vagina

**DOI:** 10.1128/MRA.00427-23

**Published:** 2023-07-25

**Authors:** Gabriela Magossi, Devin B. Holman, Kaycie N. Schmidt, Scott A. Hoselton, Samat Amat

**Affiliations:** 1 Department of Microbiological Sciences, NDSU, Fargo, North Dakota, USA; 2 Lacombe Research and Development Centre, Agriculture and Agri-Food Canada, Lacombe, Alberta, Canada; Portland State University, Portland, Oregon, USA

**Keywords:** bacteriophages, cattle

## Abstract

Here, we present the coding-complete genomes of 11 lytic bacteriophages isolated from bovine ruminal fluid and vaginal swabs that can infect the bacterial hosts *Alkalihalobacillus clausii, Bacillus safensis,* and *Escherichia coli*.

## ANNOUNCEMENT

Eleven bacteriophage*s* that can infect *Alkalihalobacillus clausii, Bacillus safensis,* and *Escherichia coli* were isolated from the ruminal fluid and vaginal swab samples of beef cattle. Cattle origin, sample collection, and bacterial host isolation were described previously (1, 2). For phage isolation, pooled ruminal fluid or vaginal swabs (*n* = 5 animals each pool) were suspended in phosphate buffered saline and centrifuged at 12,000 × *g* for 15 min at 4°C. Supernatants were passed through a 0.22-µm filter and used for a spot assay. Overnight bacterial host cultures (100 µL) were inoculated into 3 mL of brain heart infusion (BHI) soft agar (0.5% agar) which was then poured on top of BHI agar plates. Then, 10 µL of the filtered supernatant was spotted on top agar and incubated overnight at 37°C with 5% CO_2_. Areas of lysis were transferred into 1 mL of SM buffer (G-Biosciences), centrifuged at 20,000 × *g* for 1 min. The supernatant was then filtered using a 0.22-µm filter and stored at 4°C. Next, 100 µL of overnight host bacterial culture and 100 µL of phage stock were added to 3 mL of soft BHI agar, poured onto BHI plates, and incubated overnight at 37°C with 5% CO_2._ Phages were purified by two successive rounds of plating with the bacterial hosts.

Genomic DNA from the phages was extracted using the Norgen Biotek Phage DNA isolation kit (Norgen Biotek, Thorold, ON, Canada) according to manufacturer’s instructions with an additional initial step in which DNase and proteinase K were used to remove host genetic material (3). Genomic libraries for all bacteriophages (except 1RFP6A and 3RFP5C_6) were prepared using the Qiagen FX DNA library prep kit (Qiagen, Germantown, MD, USA) and sequenced on an Illumina MiSeq instrument with the MiSeq v2 reagent kit (500 cycles; Illumina, Inc, San Diego, CA, USA). Genomic DNA from 1RFP6A and 3RFP5C_6 isolates was extracted using a bead beating lysis method with the Zymo Quick-DNA Miniprep kit (Zymo Research Corporation, Irvine, CA, USA), and genomic libraries were prepared using the Illumina DNA Prep kit with UDI 10 bp indices. Libraries were sequenced on an Illumina NovaSeq 6000 instrument with an S4 flow cell (300 cycles). Quality control and adapter trimming for all genomes were performed using Trim Galore v. 0.6.10 (4) and assembled using Shovill v. 1.1.0 (https://github.com/tseemann/shovill). Qualities were assessed with QUAST (5), assemblies annotated with Pharokka v. 1.1.0 (6), and screened for virulence factors, antimicrobial resistance genes, and tRNAs with CARD (7), VFDB (8), and tRNA-scanSE v. 2.0.11 (9), respectively.

Bacteriophage genomes varied in size from 20,216 to 168,609 bp. Sequencing coverage was determined using the formula: coverage = (number of reads × read length)/genome length. None of the genomes contained CRISPRs, tmRNAs, virulence factors, or antimicrobial resistance genes. Genomes were compared to those in the GenBank database using BLASTn. Sequence similarity (% identity × % coverage) was used to determine taxonomy. The identities of ≥95%, 70%–95%, or <70% were considered as the same species, genus, or family, respectively (10) (Table 1).

**TABLE 1:** Sequencing summary and characteristics of 11 bacteriophages from ruminal fluid and vaginal swabs of healthy beef cattle

Phage	Sample type	Host	GenBank accession number	SRA accession number	Coverage	No. of reads	Length (bp)	GC content (%)	Taxonomy
1RFP6A	Ruminal fluid	*Escherichia coli*	SAMN35002581	SRR24524243	7872.907	1,217,433	46,700	44.12	*Caudoviricetes* phage
2RFP1A2_1	Ruminal fluid	*Escherichia coli* O20:H8	SAMN35002582	SRR24524242	3640.33	645,720	88,335	38.95	*Felixounavirus* sp.
2RFP1C2_AA	Ruminal fluid	*Escherichia coli* O50:H5	SAMN35002583	SRR24524240	1530.901	416,365	135,443	43.69	*Vequintavirus* sp.
2RFP1E_AA	Ruminal fluid	*Alkalihalobacillus clausii*	SAMN35002584	SRR24524239	13976.24	417,407	14,873	44.35	*Caudoviricetes* phage
2RFP1H_AA	Ruminal fluid	*Escherichia coli* O20:H8	SAMN35002585	SRR24524238	1773.773	314,631	88,335	38.94	*Felixounavirus* sp.
2RFP5B2_3	Ruminal fluid	*Bacillus safensis*	SAMN35002586	SRR24524237	6536.175	541,400	41,250	40.1	*Caudoviricetes* phage
2RFP8A_3	Ruminal fluid	*Bacillus safensis*	SAMN35002587	SRR24524236	16381.78	665,008	20,216	37.02	*Salasmaviridae* family phage
3RFP5C_2	Ruminal fluid	*Bacillus safensis*	SAMN35002588	SRR24524235	8174.731	432,243	26,332	41.06	*Herelleviridae* family phage
3RFP5E_6	Ruminal fluid	*Bacillus safensis*	SAMN35002589	SRR24524234	8794.385	4,325,964	148,554	39.04	*Siophivirus* sp.
TP167CBC_ERF3	Ruminal fluid	*Escherichia coli* O171:H25	SAMN35002591	SRR24524233	3134.37	245,897	39,069	33.94	*Tequatroviru nbeco*
TP1813CBA_EVS2	Vaginal swab	*Escherichia coli* O171:H25	SAMN35002592	SRR24524241	861.1041	291,546	168,609	35.41	*Tequatrovirus* sp.

## Data Availability

All raw genome sequences and draft genome assemblies have been deposited in the Sequence Read Archive and GenBank, respectively, under the accession numbers listed in Table 1
